# Association between total cholesterol levels and all-cause mortality among newly diagnosed patients with cancer

**DOI:** 10.1038/s41598-023-50931-6

**Published:** 2024-01-02

**Authors:** Seohyun Kim, Gyuri Kim, So Hyun Cho, Rosa Oh, Ji Yoon Kim, You-Bin Lee, Sang-Man Jin, Kyu Yeon Hur, Jae Hyeon Kim

**Affiliations:** 1 Department of Clinical Research Design and Evaluation, Samsung Advanced Institute for Health Sciences and Technology, Sungkyunkwan University, Seoul, 06355 Republic of Korea; 2 Division of Endocrinology and Metabolism, Department of Medicine, Samsung Medical Center, Sungkyunkwan University School of Medicine, 81, Irwon-ro, Gangnam-gu, Seoul, 06351 Republic of Korea

**Keywords:** Cancer epidemiology, Medical research

## Abstract

We aimed to determine the association between cholesterol values and the risk of all-cause mortality in newly diagnosed patients with cancer in a large-scale longitudinal cohort. Newly diagnosed patients with cancer were reviewed retrospectively. Cox proportional hazards regression models determined the association between baseline levels of total cholesterol (TC), triglycerides, high-density lipoprotein (HDL), and low-density lipoprotein (LDL) cholesterol and the risk of all-cause mortality. A restricted cubic spline curve was used to identify the association between total cholesterol (TC) and low-density lipoprotein (LDL) cholesterol with the risk of death on a continuous scale and to present the lowest values of lipid measurements associated with death. The median follow-up duration of the study was 5.77 years. Of the 59,217 patients with cancer, 12,624 patients were expired. The multivariable adjusted hazard ratio (aHR) for all-cause mortality in patients with cancer with 1st–5th (≤ 97 mg/dL) and 96th–100th (> 233 mg/dL) in TC levels was 1.54 (95% CI 1.43–1.66) and 1.28 (95% CI 1.16–1.41), respectively, compared to 61st–80th (172–196 mg/dL). The TC level associated with the lowest mortality risk in the multivariable model was 181 mg/dL. In comparison with LDL-C levels in the 61st–80th (115–136 mg/dL), the multivariable aHR for all-cause mortality in cancer patients with LDL-C levels in the 1st-5th (≤ 57 mg/dL) and 96th–100th (> 167 mg/dL) was 1.38 (95% CI 1.14–1.68) and 0.94 (95% CI 0.69–1.28), respectively. The 142 mg/dL of LDL cholesterol showed the lowest mortality risk. We demonstrated a U-shaped relationship between TC levels at baseline and risk of mortality in newly diagnosed patients with cancer. Low LDL levels corresponded to an increased risk of all-cause death.

## Introduction

Numerous cancers have been linked to the formation of cholesterol metabolites^1–3^. Many cohort studies have found a significant association between serum cholesterol levels and the risk of cancer incidence and recurrence in pancreas, breast and prostate cancer, with each 10 mg/dL increase in cholesterol level increasing the probability of prostate cancer recurrence by 9%^4–10^. Fat accumulation or tumor growth may result from alterations in biochemical and molecular processes that regulate lipid metabolism and cell death/proliferation^11^. The function of inappropriate lipid metabolism in the emergence of cancer has also been investigated, and troubles with the metabolism of lipids and lipoproteins brought on by metabolic syndrome and overweight/obesity have been linked to an increased risk of cancer and influence the prognosis of a patient^12–15^.

A systematic review and meta-analysis found a substantial association between serum blood total cholesterol (TC), high-density lipoprotein cholesterol (HDL-C), and overall survival among patients with cancer^16^. Recent studies have found a U-shaped association between low-density lipoprotein cholesterol (LDL-C) levels and all-cause mortality^17,18^; however, there was no substantial association between LDL-C levels and overall survival in patients with soft tissue sarcoma^19^. Previous research showed that elevated blood triglycerides (TG) levels were a poor prognostic indicator, while other research indicated no conclusive evidence of such an association^19,20^.

To the best of our knowledge, although previous studies have identified a substantial association between TC levels and mortality risk in various cancer types, there was heterogeneity among the studies (I-squared = 87.2%)^16^, and no studies identified the relationship between TC and mortality risk in large scale and all types of cancer at the first diagnosis without metastasis. Therefore, we aimed to determine the effect of TC on mortality risk in patients with cancer in a large longitudinal cohort. Furthermore, we evaluated the association of LDL-C, HDL-C, and TG with death in patients with cancer.

## Results

### Baseline characteristics of the study population

All baseline characteristics divided by centile in TC are shown in Table 1. The participants in lower centiles of TC were older, more likely to be female, had a lower BMI, higher prevalence of taking lipid-lowering agents, positive for chronic kidney disease, DM, and hypertension. Supplementary Table 1 presents all baseline characteristics by LDL-C centile and shows a similar trend to Table 1.

**Table 1:** Baseline characteristics of the study participants according to centile of the total cholesterol.

	1st–5th (≤ 97 mg/dL) (N = 3012)	6th–20th (97–128 mg/dL) (N = 9217)	21st–40th (129–151 mg/dL) (N = 12,006)	41st–60th (152–171 mg/dL) (N = 11,467)	61st–80th (172–196 mg/dL) (N = 11,903)	81st–95th (197–233 mg/dL) (N = 8690)	96th–100th (> 233 mg/dL) (N = 2922)
Age, year	63.0 ± 11.7	61.0 ± 12.1	59.4 ± 12.4	57.9 ± 12.1	56.9 ± 11.8	56.5 ± 10.9	56.1 ± 10.6
Sex							
Female	628 (20.8%)	2670 (29.0%)	4549 (37.9%)	5171 (45.1%)	6137 (51.6%)	4951 (57.0%)	1842 (63.0%)
Male	2384 (79.2%)	6547 (71.0%)	7457 (62.1%)	6296 (54.9%)	5766 (48.4%)	3739 (43.0%)	1080 (37.0%)
BMI, kg/m^2^	23.4 ± 3.3	23.7 ± 3.2	23.8 ± 3.3	23.8 ± 5.2	23.9 ± 3.2	24.2 ± 3.2	24.4 ± 3.1
Serum creatinine	1.0 ± 0.6	0.9 ± 0.6	0.9 ± 0.5	0.9 ± 0.5	0.8 ± 0.4	0.8 ± 0.3	0.8 ± 0.4
Fasting glucose, mg/dL	120.0 ± 49.4	116.2 ± 41.4	112.8 ± 37.1	110.1 ± 32.3	108.9 ± 32.1	108.2 ± 29.5	111.9 ± 36.7
ALT, U/L	31.5 ± 58.2	27.8 ± 46.0	25.5 ± 38.8	24.6 ± 36.5	24.7 ± 38.3	26.4 ± 50.4	39.4 ± 79.1
AST﻿, U/L	33.2 ± 67.4	27.9 ± 34.9	25.8 ± 32.5	24.6 ± 26.9	24.7 ± 29.9	25.4 ± 33.3	35.2 ± 56.5
eGFR, ml/min/1.73 m^﻿2 ^	87.7 ± 18.8	89.9 ± 18.2	91.6 ± 17.1	93.3 ± 16.2	94.5 ± 15.4	94.3 ± 14.5	95.2 ± 15.1
Presence of CKD							
No	2763 (91.7%)	8623 (93.6%)	11,425 (95.2%)	11,050 (96.4%)	11,569 (97.2%)	8493 (97.7%)	2854 (97.7%)
Yes	249 (8.3%)	594 (6.4%)	581 (4.8%)	417 (3.6%)	334 (2.8%)	197 (2.3%)	68 (2.3%)
Presence of DM							
No	1912 (63.5%)	6150 (66.7%)	8791 (73.2%)	9006 (78.5%)	9694 (81.4%)	7273 (83.7%)	2387 (81.7%)
Yes	1100 (36.5%)	3067 (33.3%)	3215 (26.8%)	2461 (21.5%)	2209 (18.6%)	1417 (16.3%)	535 (18.3%)
Presence of hypertension							
No	708 (23.5%)	2621 (28.4%)	3946 (32.9%)	4237 (36.9%)	4821 (40.5%)	3554 (40.9%)	1161 (39.7%)
Yes	2304 (76.5%)	6596 (71.6%)	8060 (67.1%)	7230 (63.1%)	7082 (59.5%)	5136 (59.1%)	1761 (60.3%)
History of using lipid-lowering agents							
No	2323 (77.1%)	6976 (75.7%)	9841 (82.0%)	10,030 (87.5%)	10,867 (91.3%)	8044 (92.6%)	2580 (88.3%)
Yes	689 (22.9%)	2241 (24.3%)	2165 (18.0%)	1437 (12.5%)	1036 (8.7%)	646 (7.4%)	342 (11.7%)
Smoking status							
Never	1482 (49.2%)	4860 (52.7%)	7009 (58.4%)	7127 (62.2%)	7846 (65.9%)	5915 (68.1%)	2115 (72.4%)
Ever	813 (27.0%)	2333 (25.3%)	2651 (22.1%)	2188 (19.1%)	1979 (16.6%)	1320 (15.2%)	333 (11.4%)
Current	717 (23.8%)	2024 (22.0%)	2346 (19.5%)	2152 (18.8%)	2078 (17.5%)	1455 (16.7%)	474 (16.2%)
Alcohol consumption							
Never	1300 (43.2%)	4402 (47.8%)	6283 (52.3%)	6288 (54.8%)	6889 (57.9%)	5168 (59.5%)	1805 (61.8%)
Ever	922 (30.6%)	2529 (27.4%)	2903 (24.2%)	2540 (22.2%)	2284 (19.2%)	1598 (18.4%)	434 (14.9%)
Current	790 (26.2%)	2286 (24.8%)	2820 (23.5%)	2639 (23.0%)	2730 (22.9%)	1924 (22.1%)	683 (23.4%)
Cancer types							
Gastrointestinal	1362 (45.2%)	4745 (51.5%)	5769 (48.1%)	4826 (42.1%)	4656 (39.1%)	3046 (35.1%)	872 (29.8%)
Urology	253 (8.4%)	272 (3.0%)	446 (3.7%)	474 (4.1%)	532 (4.5%)	429 (4.9%)	123 (4.2%)
Gynecology	8 (0.3%)	34 (0.4%)	45 (0.4%)	61 (0.5%)	96 (0.8%)	88 (1.0%)	29 (1.0%)
Breast	21 (0.7%)	303 (3.3%)	974 (8.1%)	1633 (14.2%)	2327 (19.5%)	2217 (25.5%)	932 (31.9%)
Hepato-pancreatobiliary	772 (25.6%)	1532 (16.6%)	1319 (11.0%)	904 (7.9%)	737 (6.2%)	439 (5.1%)	292 (10.0%)
Lung	290 (9.6%)	1768 (19.2%)	2586 (21.5%)	2486 (21.7%)	2258 (19.0%)	1434 (16.5%)	319 (10.9%)
Thyroid	16 (0.5%)	118 (1.3%)	317 (2.6%)	494 (4.3%)	649 (5.5%)	542 (6.2%)	195 (6.7%)
Others	290 (9.6%)	445 (4.8%)	550 (4.6%)	589 (5.1%)	648 (5.4%)	495 (5.7%)	160 (5.5%)

*ALT* alanine aminotransferase, *AST* aspartate aminotransferase, *BMI* body mass index, *CKD* chronic kidney disease, *DM* diabetes mellitus, *eGFR* estimated glomerular filtration rate.

### Multivariable Cox regression of all-cause mortality

**Figure 1:**
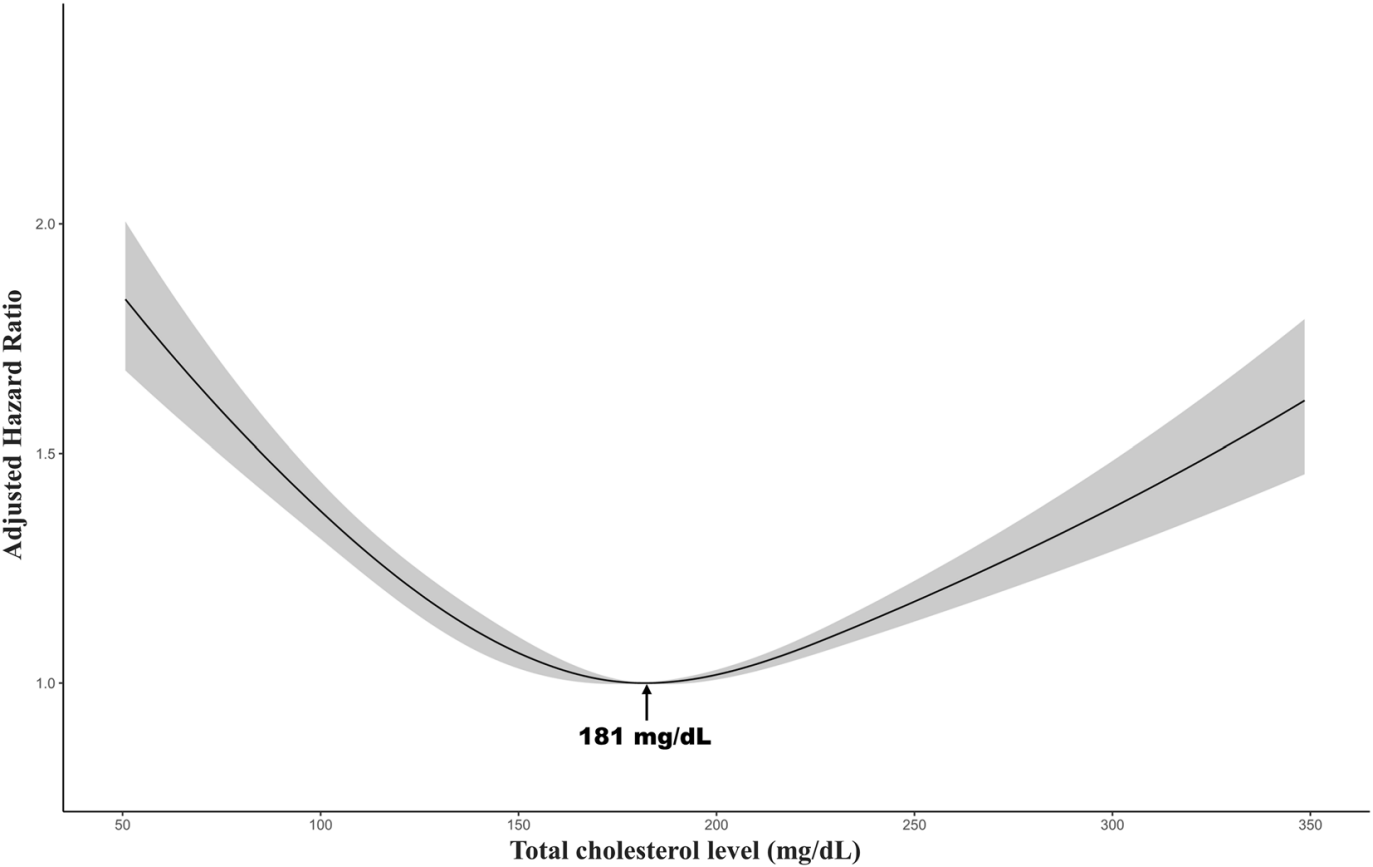
Multivariable-adjusted hazard ratios for all-cause mortality according to levels of total cholesterol on a continuous scale. Multivariable-adjusted model is adjusted for age, sex, BMI, use of lipid-lowering agents, presence of CKD, DM, and HTN, smoking status, alcohol consumption, and cancer type.

**Figure 2:**
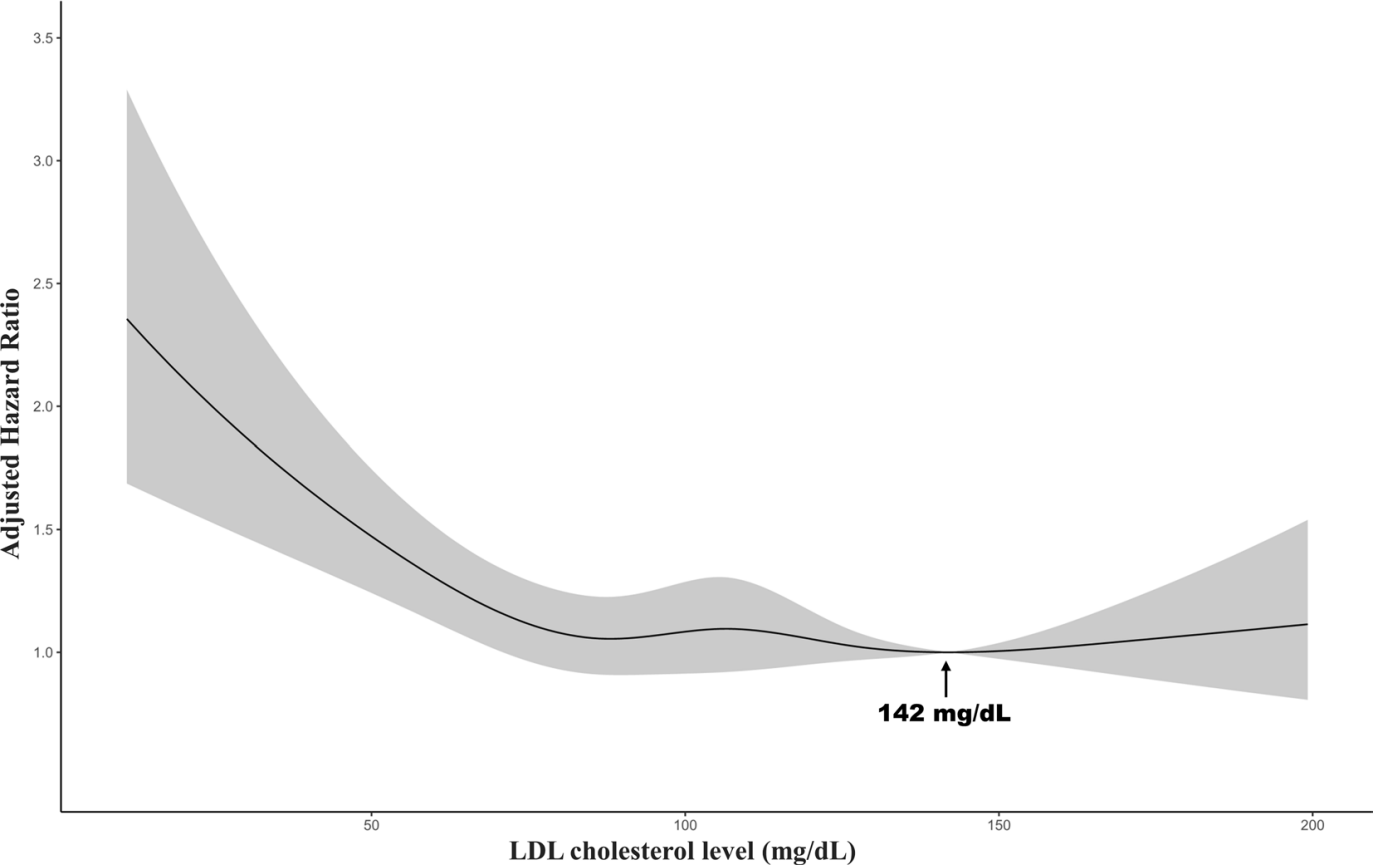
Multivariable-adjusted hazard ratios for all-cause mortality according to levels of low-density lipoprotein cholesterol on a continuous scale. Multivariable-adjusted model is adjusted for age, sex, BMI, use of lipid-lowering agents, presence of CKD, DM, and HTN, smoking status, alcohol consumption, and cancer type.

The median follow-up duration of all subjects was 5.77 years. Of the 359,380 person-years, 12,624 patients were expired. The result of multivariable Cox regression of all-cause mortality with incidence rate is described in Table 2. A U-shape association between TC level and mortality was observed in the age and sex-adjusted model and multivariable model (Table 2, Fig. 1). The multivariable aHR for all-cause mortality in patients with cancer with 1st–5th (≤ 97 mg/dL) and 96th–100th (> 233 mg/dL) in TC levels was 1.54 (95% CI 1.43–1.66) and 1.28 (95% CI 1.16–1.41), respectively, compared to 61st–80th (172–196 mg/dL). The TC level associated with the lowest mortality risk in the multivariable model was 181 mg/dL (Fig. 1). The multivariable aHR for all-cause mortality in patients with cancer with LDL-C levels in the 1st–5th (≤ 57 mg/dL) and 96th–100th (> 167 mg/dL) was 1.38 (95% CI 1.14–1.68) and 0.94 (95% CI 0.69–1.28), respectively, compared to 61st–80th (115–136 mg/dL). In the multivariable model, 142 mg/dL of LDL-C was linked with the lowest mortality risk (Fig. 2). In the multivariable analysis identifying association with HDL-C level and all-cause mortality, Q3 (50–61 mg/dL) (aHR 1.27, 95% CI 1.05–1.54), Q2 (40–50 mg/dL) (aHR 1.66, 95% CI 1.39–1.98), and Q1 (≤ 40 mg/dL) (aHR 2.10, 95% CI 1.77–2.50) were significantly associated with an increased risk of mortality compared to Q4 (> 61 mg/dL). No association was observed between TG level stratified by quartile and risk of mortality.

**Table 2:** Multivariable Cox regression for mortality by total cholesterol, LDL-C, HDL-C, and TG.

	No. of patients	Events	Person-years	Incidence rate (per 10,000 py)	Age and sex adjusted hazard ratio (95%CI)	Multivariable-adjusted hazard ratio (95%CI)	HR (95%CI)
Association between TC at cancer diagnosis and the risk of all-cause death by centile groups							
1st–5th (≤ 97 mg/dL)	3012	1143	15,924	717.8	1.79 (1.66–1.93)		1.54 (1.43–1.66)
6th–20th (97–128 mg/dL)	9217	2711	52,843	513.0	1.46 (1.38–1.55)		1.26 (1.19–1.34)
21st–40th (129–151 mg/dL)	12,006	2756	72,626	379.5	1.20 (1.13–1.27)		1.09 (1.03–1.16)
41st–60th (152–171 mg/dL)	11,467	2222	70,407	315.6	1.09 (1.03–1.16)		1.03 (0.97–1.09)
61st–80th (172–196 mg/dL)	11,903	1310	54,828	238.9	Reference		Reference
81st–95th (197–233 mg/dL)	8690	1970	74,323	265.1	0.96 (0.89–1.03)		1.02 (0.95–1.09)
96th–100th (> 233 mg/dL)	2922	512	18,428	277.8	1.20 (1.09–1.33)		1.28 (1.16–1.41)
Association between LDL-C at cancer diagnosis and the risk of all-cause death by centile groups							
1st–5th (≤ 57 mg/dL)	646	197	2,579	76.4	2.10 (1.74–2.54)		1.38 (1.14–1.68)
6th–20th (57–79 mg/dL)	1811	365	8,374	43.6	1.56 (1.33–1.83)		1.19 (1.01–1.40)
21st–40th (79–97 mg/dL)	2427	344	12,306	28.0	1.17 (1.00–1.38)		1.02 (0.87–1.20)
41st–60th (97–115 mg/dL)	2548	296	13,110	22.6	1.09 (0.92–1.28)		1.08 (0.91–1.28)
61st–80th (115–136 mg/dL)	2368	252	12,546	20.1	Reference		Reference
81st–95th (136–167 mg/dL)	1786	160	9,560	16.7	0.89 (0.73–1.08)		0.95 (0.78–1.16)
96th–100th (> 167 mg/dL)	598	48	3,142	15.3	0.89 (0.65–1.22)		0.94 (0.69–1.28)
Association between HDL-C at cancer diagnosis and the risk of all-cause death ﻿by quartile groups							
Q1 (≤ 40 mg/dL)	3270	772	14,593	529	2.65 (2.24–3.14)		2.10 (1.77–2.50)
Q2 (40–50 mg/dL)	3097	445	15,895	280	1.72 (1.44–2.05)		1.66 (1.39–1.98)
Q3 (50–61 mg/dL)	2798	266	15,087	176.3	1.24 (1.03–1.50)		1.27 (1.05–1.54)
Q4 (> 61 mg/dL)	3022	179	16,054	111.5	Reference		Reference
Association between TG at cancer diagnosis and the risk of all-cause death by quartile groups							
Q1 (≤ 73 mg/dL)	3499	487	18,784	259.3	1.02 (0.90–1.15)		0.96 (0.84–1.09)
Q2 (73–99 mg/dL)	3413	520	16,961	306.6	1.02 (0.90–1.15)		0.95 (0.83–1.07)
Q3 (99–138 mg/dL)	3440	524	17,286	303.1	1.00 (0.88–1.13)		0.93 (0.82–1.06)
Q4 (> 138 mg/dL)	3381	500	17,871	279.8	Reference		Reference

Multivariable-adjusted model was adjusted for age, sex, BMI, use of lipid-lowering agents, presence of CKD, DM, and HTN, smoking status, alcohol consumption, and cancer type.

*CKD* chronic kidney disease, *DM* diabetes mellitus, *HDL-C* high-density lipoprotein cholesterol, *HR* hazard ratio, *HTN* hypertension, *LDL-C* low-density lipoprotein cholesterol, *TG* triglyceride, *TC* total cholesterol, *CI* confidence interval.

### Sensitivity analysis

We performed a sensitivity analysis for patients with cancer who achieved NED status at the NED start date rather than continuing their follow-up to the study's objective. According to Table 3, the multivariable aHR for all-cause mortality in patients with cancer with TC levels in the first through fifth deciles (≤ 97 mg/dL) and the 96th through 100th deciles (> 233 mg/dL) was 1.60 (95% CI 1.46–2.74) and 1.35 (95% CI 1.21–1.51), respectively, compared to the 61st through 80th (172–196 mg/dL), and a U-shaped association was still observed. In terms of association between TC levels and reaching the NED status, the first through fifth deciles (≤ 97 mg/dL) was significantly associated with low probability of the NED (0.90 odds ratio, 95%CI; 0.83–0.99) compared to the 61st through 80th (172–196 mg/dL) (Supplementary Table 3). A similar relationship with the all-cause mortality was observed when limited to cancer-related mortality (Supplementary Table 4). In addition, when cut-off points for healthy people are applied, it appeared to increase mortality in cholesterol groups above 240 mg/dL (Supplementary Table 5). We found similar results to main analysis when conducting sensitivity analyses excluding people without CKD, DM, and HTN and people who died within 2 or 5 years from baseline (Supplementary Tables 6, 7, 8). When the relationship between cholesterol in the centile group of total cholesterol and death was stratified into cancer types, the relationship was more clearly observed in carcinomas with a poor prognosis such as lung cancer than in cancers with a good prognosis such as breast cancer (Supplementary Table 9).

**Table 3:** Sensitivity analysis censoring the NED by multivariable Cox regression for mortality by centile of TC.

	No. of patients	Events	Person-years	Incidence rate (per 10,000 py)	Age and sex-adjusted HR (95% CI)	Multivariable-adjusted HR (95% CI)
1st–5th (≤ 97 mg/dL)	3012	894	10,467	854.2	1.86 (1.71–2.02)	1.60 (1.46–1.74)
6th–20th (97–128 mg/dL)	9217	2051	32,517	630.7	1.52 (1.42–1.62)	1.29 (1.20–1.38)
21st–40th (129–151 mg/dL)	12,006	2030	44,129	460.0	1.20 (1.12–1.29)	1.08 (1.01–1.16)
41st–60th (152–171 mg/dL)	11,467	1690	42,565	397.0	1.13 (1.05–1.21)	1.06 (0.99–1.13)
61st–80th (172–196 mg/dL)	11,903	1466	44,807	327.2	Reference	Reference
81st–95th (197–233 mg/dL)	8690	996	33,300	299.1	0.96 (0.88–1.04)	1.03 (0.95–1.11)
96th–100th (> 233 mg/dL)	2922	410	11,228	365.1	1.25 (1.12–1.39)	1.35 (1.21–1.51)

Multivariable-adjusted model was adjusted for age, sex, BMI, use of lipid-lowering agents, presence of CKD, DM, and HTN, smoking status, alcohol consumption, and cancer type.

*CKD* chronic kidney disease, *DM* diabetes mellitus, *HR* hazard ratio, *HTN* hypertension, *NED* no evidence of disease, *TC* total cholesterol, *CI* confidence interval.

## Discussion

We showed a U-shaped association between baseline TC levels and risk of mortality in a large-scale longitudinal analysis of 59,217 patients with cancer, with low and high levels associated with an elevated risk. Low LDL-C levels (first and second centile groups) were associated with increased risk of death, but no similar risk increase was observed among patients with higher LDL-C (sixth and seventh centile groups). As expected, a decrease in HDL-C values was associated with an increased risk of death in patients with cancer. There was no significant association between TG levels by quartile and risk of mortality in patients with cancer. We demonstrated that lipid level played a comprehensive role in mortality risk in these patients. These new findings might show a clinical prognostic role of baseline lipid levels in newly diagnosed patients with cancer.

Previous systematic reviews and meta-analyses reported that high TC levels demonstrated a protective effect on the overall survival rate, and this relationship was also observed differently in our study^16,21^. Previous studies have reported that higher cholesterol levels have a protective effect on survival, and in our study, not only high cholesterol levels but also low cholesterol levels have been found to increase the risk of death. In terms of methodology, this difference may be between the results of meta-analyses of studies that focused on diverse cancer types and those produced from a single study involving various cancer types, but this requires further investigation. Moreover, previous studies reported no significant association between LDL-C levels and mortality risk in patients with cancer or significant U-shaped association in the general population; however, our study showed that low LDL-C values were associated with an increased risk of mortality in patients with cancer^16,17^. The LDL-C level with the lowest risk of mortality estimated by our study was similar to that in the general population^17^, and it is consistent with the context of previous findings that indicated TG and the risk of mortality are not significant and that the lower the HDL levels in patients with cancer, the higher the risk of death^16^.

Several mechanisms have been proposed to describe this phenomenon. In terms of reverse causation, low serum cholesterol levels can be a marker of frailty, illness, and malnutrition, which may be related to non-cardiovascular events^22,23^. The observed correlation has been explained by reverse causation, whereby undiscovered cancer lowers cholesterol levels. Our data confirmed a trend toward lower cholesterol levels in hepato-pancreatobiliary carcinoma, which is associated with relatively late detection and poor prognosis, and the probability of reaching NED in the lowest cholesterol group was significantly lower than that in the fifth quartile, indicating the possibility of reverse causality (Supplementary Tables 2 and 3)^24^. In our study, patients with the lowest levels of LDL-C were older and had more comorbidities, including diabetes, CKD, and hypertension. In our previously investigation, we identified that the presence of diabetes and chronic kidney disease increased the mortality risk in cancer patients^25^. However, sensitivity analyses, excluding patients with comorbidity or those who died within 2 or 5 years, yielded results similar to main analysis. In addition, people with dysregulated cholesterol may frequently have immune system dysfunction. Total T and CD8+ cells were considerably lower in the reduced cholesterol group compared with those in the high cholesterol group and dysregulated cholesterol homeostasis might affect pathogenesis and metastasis of malignancy by favoring cells that can resist ferroptotic cell death^26,27^. Patients with cancer with blood TC levels less than 180 mg/dL had serum interleukin-6 levels much higher than those of their respective counterparts, which were linked to the development and progression of cancer^28^. Previous studies have also discovered that the total serum antioxidant activity of the low-cholesterol group was lower than that of the high-cholesterol group, increasing their vulnerability to oxidative stress. When it comes to low LDL levels increasing the risk of death, LDL-C has been linked to lowering infectious mortality and may have the ability to prevent cancer because some cancer types can be caused by viruses. LDL-C plays a substantial role in host defense against both bacterial and viral pathogens^29–31^.

This study is the first of its kind to identify the U-shaped association between TC and all-cause mortality with long-term follow-up in a large sample size of patients with cancer. We successfully demonstrated that low LDL-C levels increased the risk of death in patients with cancer. Second, we presented the levels of TC and LDL-C that showed the lowest risk of death. Third, we performed sensitivity analysis censoring NED status during the study period instead of tracking it at the end of the study. However, our study has potential limitations. First, the study focuses on a sample of Korean individuals, and given that the demographics of a single hospital are not representative of all patients with cancer, there is a potential for selection bias. Even after performing the multivariable analysis and accounting for several confounders, it was not possible to rule out the potential of residual confounding factors, such as cancer medication or operation during follow-up. In addition, although we collected the medication information from prescription orders and self-administered questionnaires, medications received by another hospital may be excluded because we only accessed medical electronic records from our hospital.

In conclusion, in this large, long-term study of cancer patients, we demonstrated a U-shaped relationship between TC levels and the risk of mortality. Low LDL-C levels were significantly associated with an increased risk of all-cause death. A decrease in HDL-C levels was significantly associated with a higher risk of death, but there was no association between TG levels and the risk of death.

## Methods

### Study design and population

Patients with cancer older than 20 years who visited the Samsung Medical Center (SMC), Seoul, Republic of Korea, between January 2008 and December 2019 were included in this study. The study included a total of 140,133 people who had acquired a cancer diagnosis for the first time and had medical records within the TNM stage (International Classification of Disease, 10th revision; (ICD-10), C code). We included patients with cancer without metastasis and having at least 1 TC measurement at first diagnosis (N = 62,850). We excluded patients who had missing variables of BMI, laboratory measurements, medical history of hypertension, smoking status, and alcohol consumption (N = 3617) (Supplementary Fig. 1). Finally, a total of 59,217 participants were analyzed. Patients who demonstrated baseline levels of LDL, HDL, and TG were divided into the LDL (N = 12,184), HDL (N = 8867), and TG (N = 10,223) cohorts, respectively. For this investigation, all data were taken from the clinical data warehouse (CDW) DARWIN-C of SMC.

### Definition of exposure

We used the blood sample values that were closest to the date of cancer diagnosis among the measurements taken between 6 to 10 a.m. after fasting between 90 days before and after cancer diagnosis. A Modular D2400 was used to assess the plasma levels of TC, TG, HDL-C, LDL-C, and creatinine (Roche Diagnostics, Basel, Switzerland). We divided TC and LDL-C by centile and HDL and TG by quartile^17^. The date of the first cancer diagnosis was defined as the index date.

### Definition of covariates

Personal medical history was examined via electrical medical records, a self-administered questionnaire, and information on diabetes mellitus (DM), hypertension, previous smoking behavior, alcohol use, and medication. We acquired each participant's demographic, anthropometric, and laboratory information. Diabetes mellitus was defined by using the ICD-10 codes of E11–14, a self-reported history of diabetes, prescription records for anti-diabetic medications, an HbA1c of 6.5% or above, or a fasting glucose level of ≥ 126 mg/dL. To calculate the estimated glomerular filtration rate (eGFR), the Chronic Kidney Disease Epidemiology Collaboration 2021 (CKD-EPI 2021) algorithm was utilized^32^. When the eGFR was below 60 ml/min/1.73 m^2^ at the index date, chronic kidney disease (CKD) was considered as being present^33^. A BMI was calculated by dividing body weight (kg) by height (m^2^) squared. A self-reported assessment was used to gather information on alcohol use and smoking habits, which were categorized as never, ever, or current. Hypertension was defined by I10-15 in ICD-10 codes, records of anti-hypertensive drug prescriptions, a self-reported history of hypertension, or systolic or diastolic blood pressure readings at least three times greater than 140 mmHg or 90 mmHg. With the aid of medical records and an out-of-hospital drug history survey, patient history data was collected from those taking lipid-lowering agents. Eight different cancer types were created by reclassifying 24 common categories for all malignancies based on the primary site of the disease^34^. These categories included gastrointestinal (colon, rectum, stomach, esophagus, and small intestine), urologic (bladder, prostate, testis, and ureter), gynecologic (endometrial, cervix uteri, corpus uteri, and ovary), breast, hepato-pancreatobiliary (liver and intrahepatic bile duct, gallbladder and other parts of the biliary tract, and pancreas), lung, thyroid cancer, and other cancers^35,36^.

### Definition of outcomes

From the index date until the study’s termination in December 2020 or until each participant’s death (as determined by data gathered from SMC CDW death records connected to Statistics Korea), all participants were observed.

### Statistical analysis

While all continuous variables were shown as the mean and standard deviation, all categorical data were presented as percentages (SD). Using Cox proportional hazard regression models, we calculated hazard ratios (HRs) with 95% confidence intervals (CI) for all-cause mortality. Minimally adjusted model was adjusted for age and sex; multivariable-adjusted model was further adjusted for BMI, use of lipid-lowering drugs, presence of CKD, DM, and hypertension, smoking status, alcohol use, and type of malignancy. Based on multivariable-adjusted Cox regression models, the relationships between lipid levels and mortality were assessed on a continuous scale with restricted cubic spline curves. We performed a sensitivity analysis. Patients with cancer were censored at the date of no-evidence-of-disease (NED) status established by the oncologists during follow-up, rather than continuing follow-up until the termination of the investigation^37^. Also, we explored the relationship between total cholesterol and NED by multivariable logistic regression as sensitivity analysis for identifying if the group with low TC levels is less likely to reach NED than the group with high TC levels. Although the cause of death can only be identified in the case of in-hospital deaths at SMC, logistic regression analysis was performed to measure association between cholesterol levels and the risk of death from cancer.

We explored how it relates to death when we applied cutting points for healthy people^38^. We conducted sensitivity analyses excluding people without CKD, DM, and HTN and people who died within two or five years from baseline. The relationship between cholesterol and death was stratified into cancer types to explore if the aspects of the relationship were different. The statistical significance was defined as p < 0.05, and all analyses were performed using R version 2.1.3.

### Ethics approval and consent to participate

The Institutional Review Board (IRB) of Samsung Medical Center approved this study (approval no. SMC 2023-01-085). The IRB approved an informed consent exemption since the CDW of SMC de-identified and delivered all data to researchers for research. All processes were carried out in line with the Helsinki Declarations.

### Supplementary Information

Supplementary Figure 1.

Supplementary Tables.

## Data Availability

All relevant data are available in this article and supplementary files.
