# Supplementary material for: Association between total cholesterol levels and all-cause mortality among newly diagnosed patients with cancer
Source: Sci Rep. 2024 Jan 2;14:58. doi: 10.1038/s41598-023-50931-6 (PMC10761709; doi:10.1038/s41598-023-50931-6)
Supplement: Supplementary file 2 — Supplementary Tables. [file 41598_2023_50931_MOESM2_ESM.docx]

**Supplementary Table 1. Baseline characteristics of the study participants according to centile of the LDL cholesterol**

|  | 1^st^–5^th^  (≤57 mg/dL) (N=646) | | 6^th^–20^th^  (57–79 mg/dL) (N=1811) | 21^st^–40^th^  (79–97 mg/dL) (N=2427) | | 41^st^–60^th^  (97–115 mg/dL) (N=2548) | 61^st^–80^th^  (115–136 mg/dL) (N=2368) | | 81^st^–95^th^  (136–167 mg/dL) (N=1786) | 96^th^–100^th^ (>167 mg/dL) (N=598) |  |  |  |  |
| --- | --- | --- | --- | --- | --- | --- | --- | --- | --- | --- | --- | --- | --- | --- |
| Age | 66.0 ± 10.4 | | 62.6 ± 12.1 | 59.8 ± 12.4 | | 58.3 ± 11.5 | 58.0 ± 10.8 | | 57.5 ± 10.2 | 57.2 ± 9.5 |  |  |  |  |
| Sex |  | |  |  | |  |  | |  |  |  |  |  |  |
| - F | 178 (27.6%) | | 722 (39.9%) | 1144 (47.1%) | | 1320 (51.8%) | 1252 (52.9%) | | 954 (53.4%) | 365 (61.0%) |  |  |  |  |
| - M | 468 (72.4%) | | 1089 (60.1%) | 1283 (52.9%) | | 1228 (48.2%) | 1116 (47.1%) | | 832 (46.6%) | 233 (39.0%) |  |  |  |  |
| BMI | 24.1 ± 3.4 | | 24.0 ± 3.4 | 23.9 ± 3.4 | | 24.0 ± 3.2 | 24.2 ± 3.1 | | 24.6 ± 3.1 | 24.8 ± 3.0 |  |  |  |  |
| Serum Creatinine | 1.2 ± 1.3 | | 1.0 ± 1.0 | 0.9 ± 0.6 | | 0.9 ± 0.6 | 0.9 ± 0.7 | | 0.9 ± 0.5 | 0.9 ± 0.6 |  |  |  |  |
| Fasting glucose | 140.5 ± 65.4 | | 125.3 ± 52.4 | 116.3 ± 42.0 | | 111.4 ± 34.8 | 111.3 ± 37.4 | | 110.1 ± 34.2 | 112.3 ± 34.7 |  |  |  |  |
| ALT | 31.1 ± 49.2 | | 27.7 ± 41.3 | 23.4 ± 28.2 | | 23.0 ± 25.8 | 23.6 ± 31.6 | | 24.7 ± 29.7 | 29.6 ± 41.5 |  |  |  |  |
| AST | 31.9 ± 42.7 | | 27.4 ± 29.8 | 25.1 ± 35.0 | | 24.1 ± 20.0 | 24.4 ± 24.9 | | 24.1 ± 18.1 | 27.5 ± 26.3 |  |  |  |  |
| eGFR | 80.0 ± 24.2 | | 86.1 ± 22.2 | 90.4 ± 19.3 | | 92.0 ± 17.5 | 92.6 ± 16.4 | | 93.0 ± 15.3 | 92.5 ± 16.9 |  |  |  |  |
| Presence of CKD |  | |  |  | |  |  | |  |  |  |  |  |  |
| - No | 535 (82.8%) | | 1602 (88.5%) | 2251 (92.7%) | | 2417 (94.9%) | 2278 (96.2%) | | 1737 (97.3%) | 577 (96.5%) |  |  |  |  |
| - Yes | 111 (17.2%) | | 209 (11.5%) | 176 (7.3%) | | 131 (5.1%) | 90 (3.8%) | | 49 (2.7%) | 21 (3.5%) |  |  |  |  |
| Presence of DM |  | |  |  | |  |  | |  |  |  |  |  |  |
| - No | 242 (37.5%) | | 978 (54.0%) | 1658 (68.3%) | | 1947 (76.4%) | 1892 (79.9%) | | 1447 (81.0%) | 480 (80.3%) |  |  |  |  |
| - Yes | 404 (62.5%) | | 833 (46.0%) | 769 (31.7%) | | 601 (23.6%) | 476 (20.1%) | | 339 (19.0%) | 118 (19.7%) |  |  |  |  |
| Presence of hypertension | |  | | |  | | |  | | |  |  |  |  |
| - No | 106 (16.4%) | | 436 (24.1%) | 806 (33.2%) | | 955 (37.5%) | 916 (38.7%) | | 695 (38.9%) | 245 (41.0%) |  |  |  |  |
| - Yes | 540 (83.6%) | | 1375 (75.9%) | 1621 (66.8%) | | 1593 (62.5%) | 1452 (61.3%) | | 1091 (61.1%) | 353 (59.0%) |  |  |  |  |
| History of using lipid-lowering agents | |  | | |  | | |  | | |  |  |  |  |
| - No | 332 (51.4%) | | 1063 (58.7%) | 1513 (62.3%) | | 1663 (65.3%) | 1558 (65.8%) | | 1160 (64.9%) | 408 (68.2%) |  |  |  |  |
| - Yes | 164 (25.4%) | | 417 (23.0%) | 483 (19.9%) | | 478 (18.8%) | 378 (16.0%) | | 306 (17.1%) | 87 (14.5%) |  |  |  |  |
| Smoking status |  | |  |  | |  |  | |  |  |  |  |  |  |
| - Never | 346 (53.6%) | | 1022 (56.4%) | 1361 (56.1%) | | 1510 (59.3%) | 1381 (58.3%) | | 1052 (58.9%) | 373 (62.4%) |  |  |  |  |
| - Ever | 171 (26.5%) | | 401 (22.1%) | 529 (21.8%) | | 459 (18.0%) | 428 (18.1%) | | 284 (15.9%) | 86 (14.4%) |  |  |  |  |
| - Current | 129 (20.0%) | | 388 (21.4%) | 537 (22.1%) | | 579 (22.7%) | 559 (23.6%) | | 450 (25.2%) | 139 (23.2%) |  |  |  |  |
| Alcohol consumption | |  | | |  | | |  | | |  |  |  |  |
| - Never | 246 (38.1%) | | 886 (48.9%) | 1667 (68.7%) | | 2028 (79.6%) | 2031 (85.8%) | | 1547 (86.6%) | 458 (76.6%) |  |  |  |  |
| - Ever | 150 (23.2%) | | 331 (18.3%) | 431 (17.8%) | | 407 (16.0%) | 432 (18.2%) | | 320 (17.9%) | 103 (17.2%) |  |  |  |  |
| - Current | 400 (61.9%) | | 925 (51.1%) | 760 (31.3%) | | 520 (20.4%) | 337 (14.2%) | | 239 (13.4%) | 140 (23.4%) |  |  |  |  |
| Cancer types |  | |  |  | |  |  | |  |  |  |  |  |  |
| Gastrointestinal | 181 (28.0%) | | 412 (22.7%) | 571 (23.5%) | | 549 (21.5%) | 527 (22.3%) | | 415 (23.2%) | 153 (25.6%) |  |  |  |  |
| Urology | 58 (9.0%) | | 201 (11.1%) | 214 (8.8%) | | 233 (9.1%) | 216 (9.1%) | | 166 (9.3%) | 50 (8.4%) |  |  |  |  |
| Gynecology | 5 (0.8%) | | 13 (0.7%) | 20 (0.8%) | | 32 (1.3%) | 21 (0.9%) | | 23 (1.3%) | 5 (0.8%) |  |  |  |  |
| Breast | 40 (6.2%) | | 309 (17.1%) | 604 (24.9%) | | 743 (29.2%) | 732 (30.9%) | | 572 (32.0%) | 208 (34.8%) |  |  |  |  |
| Hepato-Pancreatobiliary | 114 (17.6%) | | 141 (7.8%) | 121 (5.0%) | | 87 (3.4%) | 70 (3.0%) | | 54 (3.0%) | 17 (2.8%) |  |  |  |  |
| Lung | 189 (29.3%) | | 539 (29.8%) | 649 (26.7%) | | 609 (23.9%) | 509 (21.5%) | | 323 (18.1%) | 91 (15.2%) |  |  |  |  |
| Thyroid | 4 (0.6%) | | 68 (3.8%) | 97 (4.0%) | | 114 (4.5%) | 123 (5.2%) | | 106 (5.9%) | 34 (5.7%) |  |  |  |  |
| Others | 55 (8.5%) | | 128 (7.1%) | 151 (6.2%) | | 181 (7.1%) | 170 (7.2%) | | 127 (7.1%) | 40 (6.7%) |  |  |  |  |

*Abbreviations: ALT=alanine aminotransferase; AST=aspartate aminotransferase; BMI=body mass index; CKD=chronic kidney disease; DM=diabetes mellitus; eGFR=estimated glomerular filtration rate; F=female; M=male

**Supplementary Table 2. Baseline mean of the lipid measurements according to the cancer type**

|  | **Gastrointesti-nal** | **Urology** | **Gynecology** | **Breast** | **Hepato-Pancreatobilia-ry** | **Lung** | **Thyroid** | **Others** | **P value** |
| --- | --- | --- | --- | --- | --- | --- | --- | --- | --- |
| TC | (N=25276) | (N=2529) | (N=361) | (N=8407) | (N=5995) | (N=11141) | (N=2331) | (N=3177) |  |
|  | 156.9 ± 39.4 | 159.8 ± 49.9 | 178.5 ± 42.2 | 187.6 ± 36.7 | 147.7 ± 56.9 | 160.8 ± 36.1 | 181.7 ± 36.5 | 160.0 ± 46.4 | <0.001 |
| LDL-C | (N=2808) | (N=1138) | (N=119) | (N=3208) | (N=604) | (N=2909) | (N=546) | (N=852) |  |
|  | 107.9 ± 35.4 | 106.8 ± 33.0 | 112.6 ± 32.3 | 116.1 ± 31.6 | 90.7 ± 38.9 | 102.3 ± 31.7 | 116.4 ± 33.0 | 107.8 ± 34.2 | <0.001 |
| HDL-C | (N=2805) | (N=1140) | (N=119) | (N=3211) | (N=604) | (N=2910) | (N=546) | (N=852) |  |
|  | 46.4 ± 15.5 | 50.6 ± 14.0 | 58.3 ± 16.9 | 61.8 ± 15.6 | 44.2 ± 17.8 | 45.7 ± 14.0 | 54.6 ± 16.1 | 50.5 ± 15.5 | <0.001 |
| TG | (N=3253) | (N=1461) | (N=122) | (N=3223) | (N=998) | (N=2998) | (N=577) | (N=1101) |  |
|  | 125.2 ± 81.1 | 116.1 ± 81.4 | 106.9 ± 67.3 | 104.4 ± 61.7 | 109.9 ± 66.6 | 112.8 ± 57.5 | 135.2 ± 118.7 | 142.6 ± 139.2 | <0.001 |

*Abbreviations: HDL-C=high-density lipoprotein cholesterol; LDL-C=low-density lipoprotein cholesterol; TG=Triglyceride; TC=total cholesterol;

**Supplementary Table 3. Sensitivity analysis of association between TC and NED status**

|  | No. of Patients | Events (%) | Age and sex-adjusted  OR  (95% CI) | Multivariable-adjusted  OR  (95% CI) |
| --- | --- | --- | --- | --- |
| 1^st^–5^th^  (≤97 mg/dL) | 3,012 | 1156 (38.4%) | 0.71 (0.65-0.77) | 0.90 (0.83-0.99) |
| 6^th^–20^th^  (97–128 mg/dL) | 9,217 | 4235 (45.9%) | 0.93 (0.88-0.99) | 1.04 (0.98-1.10) |
| 21^st^–40^th^ (129–151 mg/dL) | 12,006 | 5780 (48.1%) | 0.99 (0.94-1.04) | 1.03 (0.97-1.08) |
| 41^st^–60^th^ (152–171 mg/dL) | 11,467 | 5571 (48.6%) | 0.97 (0.92-1.02) | 0.99 (0.94-1.05) |
| 61^st^–80^th^ (172–196 mg/dL) | 11,903 | 5930 (49.8%) | Reference | Reference |
| 81^st^–95^th^ (197–233 mg/dL) | 8,690 | 4276 (49.2%) | 0.96 (0.91-1.02) | 0.96 (0.91-1.02) |
| 96^th^–100^th^ (>233 mg/dL) | 2,922 | 1419 (48.6%) | 0.93 (0.86-1.01) | 0.97 (0.90-1.06) |

*Multivariable-adjusted model was adjusted for age, sex, BMI, use of lipid-lowering agents, presence of CKD, DM, and HTN, smoking status, alcohol consumption, and cancer type.

*Abbreviations: CKD=chronic kidney disease; DM=diabetes mellitus; HTN=hypertension; OR=odds ratio; NED=No evidence of disease; TC=total cholesterol; CI=confidence interval

**Supplementary Table 4. Sensitivity analysis of cancer-specific mortality**

|  | No. of Patients | Events (%) | Age and sex-adjusted  OR  (95% CI) | Multivariable-adjusted  OR  (95% CI) |
| --- | --- | --- | --- | --- |
| 1^st^–5^th^  (≤97 mg/dL) | 3,012 | 444 (14.7%) | 1.68 (1.48-1.90) | 1.45 (1.37-1.65) |
| 6^th^–20^th^  (97–128 mg/dL) | 9,217 | 1049 (11.4%) | 1.36 (1.23-1.50) | 1.16 (1.11-1.28) |
| 21^st^–40^th^ (129–151 mg/dL) | 12,006 | 1057 (8.8%) | 1.12 (1.02-1.23) | 1.00 (0.94-1.11) |
| 41^st^–60^th^ (152–171 mg/dL) | 11,467 | 911  (7.9%) | 1.09 (0.99-1.20) | 1.02 (0.94-1.13) |
| 61^st^–80^th^ (172–196 mg/dL) | 11,903 | 820  (6.9%) | 1.02 (0.91-1.14) | 1.10 (0.98-1.21) |
| 81^st^–95^th^ (197–233 mg/dL) | 8,690 | 576  (6.6%) | Reference | Reference |
| 96^th^–100^th^ (>233 mg/dL) | 2,922 | 211  (7.2%) | 1.19 (1.01-1.39) | 1.26 (1.07-1.48) |

*Multivariable-adjusted model was adjusted for age, sex, BMI, use of lipid-lowering agents, presence of CKD, DM, and HTN, smoking status, alcohol consumption, and cancer type.

*Abbreviations: CKD=chronic kidney disease; DM=diabetes mellitus; HTN=hypertension; OR=odds ratio; NED=No evidence of disease; TC=total cholesterol; CI=confidence interval

**Supplementary Table 5. Sensitivity analysis using cut-off value of TC for healthy people**

|  | No. of Patients | Events | Person-years | Incidence Rate  (per 10,000 py) | Age and sex-adjusted  HR  (95% CI) | Multivariable-adjusted  HR  (95% CI) |
| --- | --- | --- | --- | --- | --- | --- |
| Optimal  (<200 mg/dL) | 3,012 | 10950 | 292,649 | 374.2 | Ref | Ref |
| Borderline  (200-240 mg/dL) | 9,217 | 1260 | 52,528 | 239.9 | 0.81 (0.76-0.86) | 0.94 (0.88-1.00) |
| High  (≥240 mg/dL) | 2,922 | 414 | 14,203 | 291.5 | 1.05 (0.95-1.16) | 1.19 (1.08-1.31) |

*Multivariable-adjusted model was adjusted for age, sex, BMI, use of lipid-lowering agents, presence of CKD, DM, and HTN, smoking status, alcohol consumption, and cancer type.

*Abbreviations: CKD=chronic kidney disease; DM=diabetes mellitus; HR=hazard ratio; HTN=hypertension; NED=No evidence of disease; TC=total cholesterol; CI=confidence interval

**Supplementary Table 6. Sensitivity analysis excluding people with CKD, DM, and HTN**

|  | No. of Patients | Events | Person-years | Incidence Rate  (per 10,000 py) | Age and sex-adjusted  HR  (95% CI) | Multivariable-adjusted  HR  (95% CI) |
| --- | --- | --- | --- | --- | --- | --- |
| 1st-5th  (≤97 mg/dL) | 543 | 182 | 3300 | 551.6 | 2.17  (1.83-2.58) | 1.87  (1.56-2.23) |
| 6th-20th  (97–128 mg/dL) | 2140 | 496 | 14350 | 345.6 | 1.67  (1.47-1.90) | 1.45  (1.27-1.65) |
| 21st-40th (129–151 mg/dL) | 3378 | 552 | 22393 | 246.5 | 1.31  (1.16-1.48) | 1.17  (1.04-1.33) |
| 41st-60th (152–171 mg/dL) | 3740 | 479 | 24245 | 197.6 | 1.13  (1.00-1.29) | 1.05  (0.93-1.20) |
| 61st-80th (172–196 mg/dL) | 4350 | 477 | 28351 | 168.2 | Ref | Ref |
| 81st-95th (197–233 mg/dL) | 3226 | 345 | 20693 | 166.7 | 1.00  (0.87-1.14) | 1.08  (0.94-1.24) |
| 96th-100th (>233 mg/dL) | 1048 | 106 | 6972 | 152.0 | 0.95  (0.77-1.17) | 1.06  (0.86-1.31) |

*Multivariable-adjusted model was adjusted for age, sex, BMI, use of lipid-lowering agents, smoking status, alcohol consumption, and cancer type.

*Abbreviations: CKD=chronic kidney disease; DM=diabetes mellitus; HR=hazard ratio; HTN=hypertension; NED=No evidence of disease; TC=total cholesterol; CI=confidence interval

**Supplementary Table 7. Sensitivity analysis excluding people with death within two years from baseline**

|  | No. of Patients | Events | Person-years | Incidence Rate  (per 10,000 py) | Age and sex-adjusted  HR  (95% CI) | Multivariable-adjusted  HR  (95% CI) |
| --- | --- | --- | --- | --- | --- | --- |
| 1st-5th  (≤97 mg/dL) | 2426 | 557 | 15340 | 363.1 | 1.54 (1.39-1.70) | 1.36 (1.23-1.51) |
| 6th-20th  (97–128 mg/dL) | 7981 | 1475 | 51526 | 286.3 | 1.38 (1.28-1.49) | 1.22 (1.13-1.32) |
| 21st-40th (129–151 mg/dL) | 10863 | 1613 | 71393 | 225.9 | 1.18 (1.10-1.27) | 1.09 (1.01-1.18) |
| 41st-60th (152–171 mg/dL) | 10571 | 1326 | 69431 | 191.0 | 1.08 (1.00-1.17) | 1.03 (0.95-1.11) |
| 61st-80th (172–196 mg/dL) | 11131 | 1198 | 73459 | 163.1 | 1.00 (0.91-1.09) | 1.04 (0.95-1.14) |
| 81st-95th (197–233 mg/dL) | 8223 | 843 | 54317 | 155.2 | 1.10 (0.97-1.25) | 1.17 (1.03-1.33) |
| 96th-100th (>233 mg/dL) | 2703 | 293 | 18199 | 161.0 | 0.95  (0.77-1.17) | 1.06  (0.86-1.31) |

*Multivariable-adjusted model was adjusted for age, sex, BMI, use of lipid-lowering agents, presence of CKD, DM, and HTN, smoking status, alcohol consumption, and cancer type.

*Abbreviations: CKD=chronic kidney disease; DM=diabetes mellitus; HR=hazard ratio; HTN=hypertension; NED=No evidence of disease; TC=total cholesterol; CI=confidence interval

**Supplementary Table 8. Sensitivity analysis excluding people with death within five years from baseline**

|  | No. of Patients | Events | Person-years | Incidence Rate  (per 10,000 py) | Age and sex-adjusted  HR  (95% CI) | Multivariable-adjusted  HR  (95% CI) |
| --- | --- | --- | --- | --- | --- | --- |
| 1st-5th  (≤97 mg/dL) | 2075 | 206 | 14214 | 144.9 | 1.55 (1.31-1.83) | 1.48 (1.25-1.76) |
| 6th-20th  (97–128 mg/dL) | 7030 | 524 | 48458 | 108.1 | 1.33 (1.17-1.51) | 1.24 (1.09-1.41) |
| 21st-40th (129–151 mg/dL) | 9890 | 640 | 68202 | 93.8 | 1.22 (1.08-1.38) | 1.16 (1.03-1.31) |
| 41st-60th (152–171 mg/dL) | 9776 | 531 | 66816 | 79.5 | 1.11 (0.98-1.26) | 1.07 (0.95-1.21) |
| 61st-80th (172–196 mg/dL) | 10400 | 467 | 71086 | 65.7 | Ref | Ref |
| 81st-95th (197–233 mg/dL) | 7722 | 342 | 52691 | 64.9 | 1.02 (0.89-1.17) | 1.03 (0.90-1.19) |
| 96th-100th (>233 mg/dL) | 2506 | 96 | 17571 | 54.6 | 0.88 (0.70-1.09) | 0.90 (0.72-1.12) |

*Multivariable-adjusted model was adjusted for age, sex, BMI, use of lipid-lowering agents, presence of CKD, DM, and HTN, smoking status, alcohol consumption, and cancer type.

*Abbreviations: CKD=chronic kidney disease; DM=diabetes mellitus; HR=hazard ratio; HTN=hypertension; NED=No evidence of disease; TC=total cholesterol; CI=confidence interval

**Supplementary Table 9. Sensitivity analysis in specific cancer type**

|  | Gastrointestinal  (aHR, 95%CI) | Breast  (aHR, 95%CI) | Hepato-  Pancreatobiliary  (aHR, 95%CI) | Lung  (aHR, 95%CI) |
| --- | --- | --- | --- | --- |
| N of patients | 25,276 | 8,407 | 5,995 | 11,141 |
| 1st-5th  (≤97 mg/dL) | 1.96 (1.74-2.20) | 1.79 (0.44-7.27) | 1.25 (1.07-1.45) | 1.34 (1.12-1.61) |
| 6th-20th  (97–128 mg/dL) | 1.52 (1.38-1.68) | 1.30 (0.81-2.09) | 0.98 (0.85-1.12) | 1.11 (0.99-1.23) |
| 21st-40th (129–151 mg/dL) | 1.21 (1.10-1.33) | 1.13 (0.81-1.58) | 0.92 (0.79-1.06) | 1.01 (0.92-1.12) |
| 41st-60th (152–171 mg/dL) | 1.09 (0.98-1.21) | 1.06 (0.80-1.40) | 0.93 (0.80-1.08) | 0.97 (0.87-1.07) |
| 61st-80th (172–196 mg/dL) | Ref | Ref | Ref | Ref |
| 81st-95th (197–233 mg/dL) | 1.01 (0.90-1.15) | 1.06 (0.82-1.36) | 1.19 (0.99-1.41) | 1.01 (0.89-1.14) |
| 96th-100th (>233 mg/dL) | 1.10 (0.91-1.34) | 0.89 (0.63-1.25) | 1.72 (1.43-2.07) | 1.15 (0.93-1.41) |

* aHR was adjusted for age, sex, BMI, use of lipid-lowering agents, presence of CKD, DM, and HTN, smoking status, alcohol consumption.

*Abbreviations: CKD=chronic kidney disease; DM=diabetes mellitus; aHR=adjusted hazard ratio; HTN=hypertension; NED=No evidence of disease; TC=total cholesterol; CI=confidence interval

**Supplementary Figure 1. Flow chart**

**
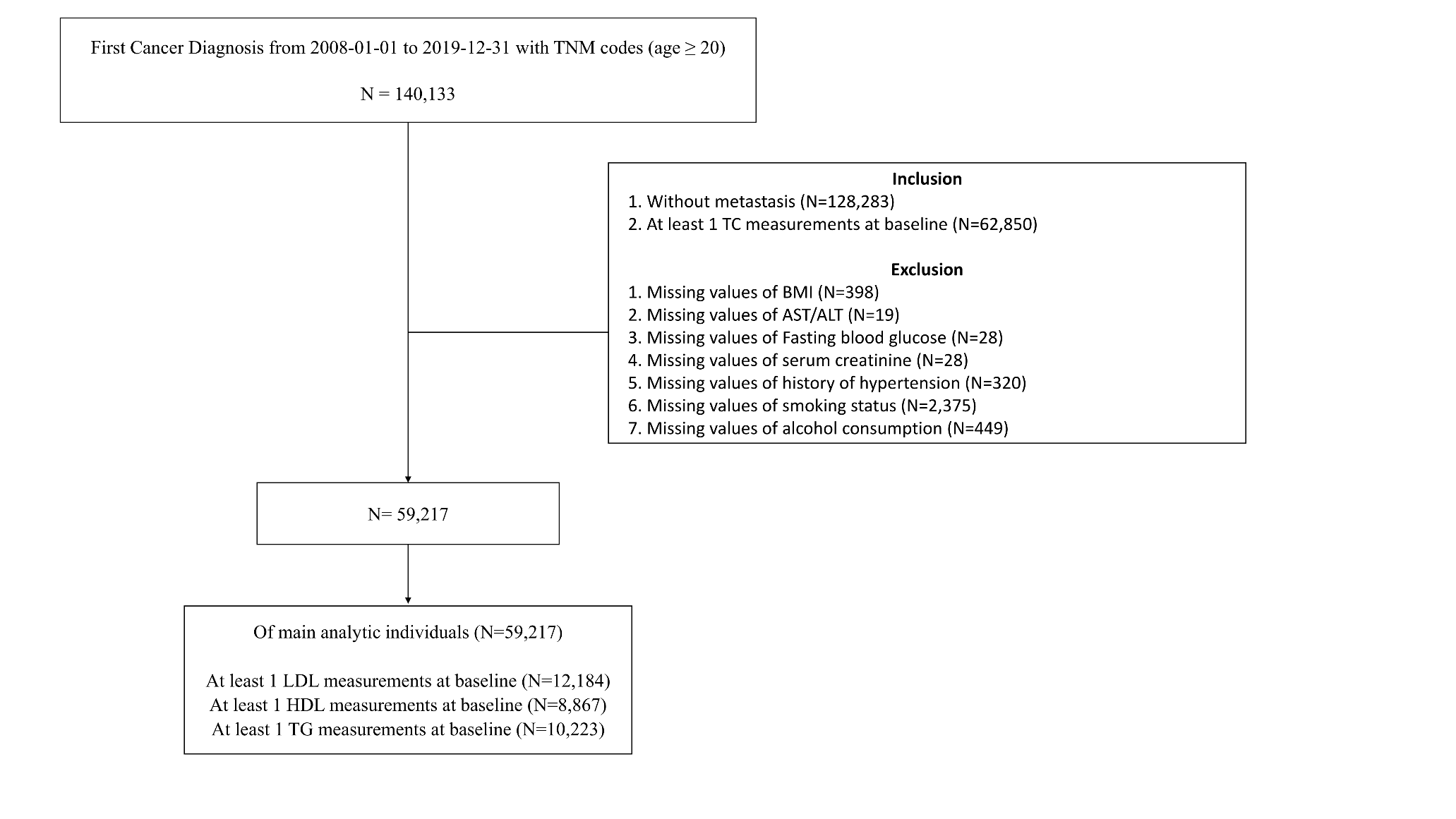
**
